# Taguchi-Based Optimization of FDM Parameters for Sub-150 µm Microchannels: Comparative Study of PETG and TPU

**DOI:** 10.3390/mi16101079

**Published:** 2025-09-24

**Authors:** Khadija Bekkay Haouari, Hicham Mastouri, Mohamed Amine Daoud, Chouaib Ennawaoui, Mustapha Ouardouz

**Affiliations:** 1 Faculty of Sciences and Techniques of Tangier (FSTT), University Abdelmalek Essaâdi, Tangier 90000, Morocco; mohamedamine.daouad@etu.uae.ac.ma (M.A.D.); mouardouz@uae.ac.ma (M.O.); 2 Energy4Water Research Center (E4W), University Mohammed VI Polytechnic (UM6P), Benguerir 43150, Morocco; hicham.mastouri@um6p.ma (H.M.); chouaib.enna@gmail.com (C.E.); 3 Laboratory of Engineering Sciences for Energy, National School of Applied Sciences, Chouaib Doukkali University, El Jadida 24000, Morocco

**Keywords:** fused deposition modeling (FDM), microfluidics, polyethylene terephthalate glycol (PETG), thermoplastic polyurethane (TPU), Taguchi method, process optimization

## Abstract

The fabrication of microfluidic components using low-cost Fused Deposition Modeling (FDM) presents an attractive alternative to conventional manufacturing methods, yet achieving microscale dimensional accuracy remains a significant challenge. This study investigates the influence of five key FDM parameters (nozzle temperature, bed temperature, printing speed, flow rate, and infill overlap) on the dimensional accuracy of microchannels printed with PETG and TPU filaments. A Taguchi L27 orthogonal array was employed to systematically evaluate the effects of these parameters on width and depth deviations across sub-millimeter microchannel geometries. Results show that for PETG, optimal dimensional fidelity was achieved at 240 °C nozzle temperature, 70 °C bed temperature, 30 mm/s speed, 100% flow rate, and 15% overlap, enabling reliable channel widths down to 100 µm. TPU exhibited greater variability due to its elasticity, with optimal settings found at 220 °C, 60 °C bed temperature, 30 mm/s, 100% flow rate, and 25% overlap. Signal-to-noise ratio and ANOVA analyses revealed flow rate and printing speed as dominant factors for both materials. The findings provide a reproducible optimization framework for microscale FDM fabrication and highlight material-specific process sensitivities critical to functional microfluidic device performance.

## 1. Introduction

Microfluidic technologies have gained substantial traction in the past two decades as powerful platforms for miniaturized fluid manipulation. Their application spans diverse domains, including biomedical diagnostics, drug delivery, chemical synthesis, and environmental sensing [1,2]. The integration of multiple fluidic operations within compact, chip-scale systems enables high-throughput analysis [3], low reagent consumption [4], and portable point-of-care solutions [5], offering transformative potential for healthcare and scientific research alike [6]. However, the widespread deployment of microfluidic systems continues to be hindered by challenges associated with their fabrication. Conventional manufacturing methods such as photolithography [7], soft lithography [8], and hot embossing [9], though precise, are typically time-consuming, require cleanroom infrastructure, and are often cost-prohibitive for iterative prototyping or decentralized use [10]. Beyond these cleanroom-dependent approaches, several other techniques have also been explored for microfluidic channel fabrication. Injection molding and hot embossing, for instance, can achieve resolutions on the order of 30 µm and are widely used for mass production of polymer-based microdevices [11,12]. However, their reliance on costly molds and specialized equipment renders them impractical for rapid prototyping or small batch fabrication. Laser cutting represents another accessible option and provides high precision in two-dimensional patterning, yet it is limited in its capacity to generate complex three-dimensional channel architectures [13,14]. Collectively, while these methods are capable of producing highly accurate features, they generally lack the flexibility, speed, and affordability required for iterative design in research and decentralized applications.

To address these limitations, additive manufacturing (AM), particularly Fused Deposition Modeling (FDM), has emerged as a promising alternative for the rapid and affordable fabrication of microfluidic devices [15]. FDM operates by extruding thermoplastic filaments layer by layer, enabling tool-free fabrication with wide material availability [16]. Its accessibility through low-cost desktop printers and the capacity for rapid iteration make it appealing for research and prototyping. Nonetheless, the inherent limitations of FDM, such as limited resolution, anisotropic properties, and poor surface finish, pose significant barriers to its application in microfluidics, where feature dimensions often fall below 500 µm and demand strict dimensional accuracy and reproducibility [17,18,19].

A major challenge lies in the control of process parameters that directly affect the deposition quality and geometry of printed features [20,21]. Parameters such as nozzle temperature, bed temperature, print speed, flow rate, and infill overlap influence not only the extrusion behavior and cooling dynamics but also the fidelity of microscale structures, particularly open-channel geometries [22,23,24,25]. Compounding these difficulties is the strong dependence of FDM behavior on the thermomechanical properties of the filament material. Materials such as Polyethylene Terephthalate Glycol (PETG) and Thermoplastic Polyurethane (TPU) are frequently used in low-cost FDM systems due to their availability and functional properties [26,27,28,29]. PETG, a semi-rigid polymer, offers favorable thermal stability and transparency, making it a candidate for rigid microfluidic components [30,31]. TPU, on the other hand, is a highly elastic material suited for flexible or stretchable microfluidic applications [32,33]. However, its softness and melt viscosity make dimensional control more challenging compared to rigid polymers.

While several studies have explored the effect of process parameters on macroscopic FDM part quality [34,35,36,37,38], relatively few have investigated parameter optimization for microscale structures or compared the performance of different polymer types under identical microfabrication constraints. Moreover, most existing work employs one-factor-at-a-time approaches, which are inefficient and fail to capture parameter interactions or process robustness. There is thus a critical need for a systematic and statistically rigorous optimization framework that can evaluate multiple parameters simultaneously and provide insight into both accuracy and variability of printed microfluidic features.

In this study, the Taguchi method, a robust statistical design of experiments (DOE) framework, is applied to optimize FDM process parameters for the fabrication of open microchannels with PETG and TPU. Using an L27 orthogonal array, the effects of five parameters on width and depth deviations in sub-millimeter channels are systematically explored. Statistical analyses, including signal-to-noise (*S*/*N*) ratios and analysis of variance (ANOVA), are employed to identify significant factors and determine optimal processing conditions. The overarching aim is to establish a reproducible framework that enhances microscale dimensional accuracy in low-cost FDM while enabling a comparative assessment of PETG and TPU.

Unlike most prior studies that focused on one-factor-at-a-time variations or a limited subset of parameters, this work systematically investigates the simultaneous influence of five critical FDM parameters (nozzle temperature, bed temperature, printing speed, flow rate, and infill overlap) on microchannel fidelity. In particular, flow rate and infill overlap, rarely addressed in microfluidic contexts despite their strong impact on channel sealing and leakage prevention, are rigorously incorporated into the optimization framework. This broader parameter space enables the identification of interactions and trade-offs that cannot be captured through conventional two-parameter studies.

Furthermore, the present work is distinguished by its focus on multi-material microfluidics, combining a rigid transparent polymer (PETG) with a flexible elastomer (TPU). While PETG provides thermal stability and dimensional accuracy for rigid channels, TPU introduces elasticity that is advantageous for flexible or wearable devices. By explicitly targeting both materials within the same statistically rigorous framework, this study represents a systematic attempt to define material-specific processing windows for hybrid rigid-flexible microfluidic devices fabricated by low-cost FDM. Notably, this optimization enables the reproducible fabrication of sub-150 µm microchannels, with feature sizes as small as 100 µm, which defines a practical resolution threshold for accessible FDM-based microfluidics.

The findings of this work aim to advance additive microfabrication by establishing reliable parameter-performance relationships for low-cost FDM in microfluidics. Beyond identifying optimal settings for PETG and TPU, the results provide material-specific insights that can guide the design of rigid and flexible microfluidic components. This foundation supports the broader objective of translating affordable FDM into a reproducible platform for microchannel fabrication, paving the way for its use in practical device-oriented applications.

## 2. Materials and Methods

### 2.1. Materials Selection

Material selection plays a critical role in determining the fidelity, repeatability, and functional integrity of microfluidic structures fabricated via Fused Deposition Modeling (FDM) [39,40]. This study focuses on two widely used thermoplastics, Thermoplastic Polyurethane (TPU) and Polyethylene Terephthalate Glycol (PETG), selected for their commercial availability in filament form, compatibility with low-cost FDM printers, and contrasting thermomechanical profiles that are representative of flexible and semi-rigid microfluidic applications.

TPU is an elastomeric polymer known for its high elongation at break and excellent mechanical flexibility, making it suitable for the fabrication of soft and deformable microfluidic systems. Its low glass transition temperature (~−30 °C) and high melt viscosity, however, present significant challenges during extrusion [27,41]. Elastic deformation of the filament within the feeding system can lead to flow instability, under-extrusion, and inconsistent layer formation, particularly at high printing speeds. Additionally, TPU’s poor dimensional stability often results in over-extruded or distorted features at the microscale. Nevertheless, its slow solidification promotes strong interlayer bonding, which supports structural robustness in flexible microchannel designs [42].

PETG, by contrast, is a semi-rigid thermoplastic with a higher glass transition temperature (~80 °C) and lower melt viscosity, offering more predictable flow behavior and improved dimensional control [43]. It combines ease of printing with moderate rigidity and optical transparency, characteristics desirable in prototyping rigid microfluidic components [44]. PETG exhibits strong interlayer adhesion and reduced warping compared to other common FDM materials [45]. However, it is susceptible to stringing and over-extrusion in fine-feature regions if flow rate and retraction settings are not carefully calibrated.

The selection of TPU and PETG enables a comparative investigation of process behavior across soft and rigid materials under identical FDM conditions. This comparison supports the development of material-specific optimization strategies for achieving reliable microchannel geometries in single-material microfluidic applications. It also lays the groundwork for future integration of multi-material printing where both mechanical flexibility and structural stability may be required within a single device.

### 2.2. Test Part Design and Printing Process

To evaluate the influence of FDM parameters on the dimensional accuracy of microscale features, a standardized test part was developed featuring a series of open rectangular microchannels with systematically decreasing dimensions. The design objective was to identify the minimum reliably printable and measurable channel size for each material under various process conditions.

**Figure 1:**
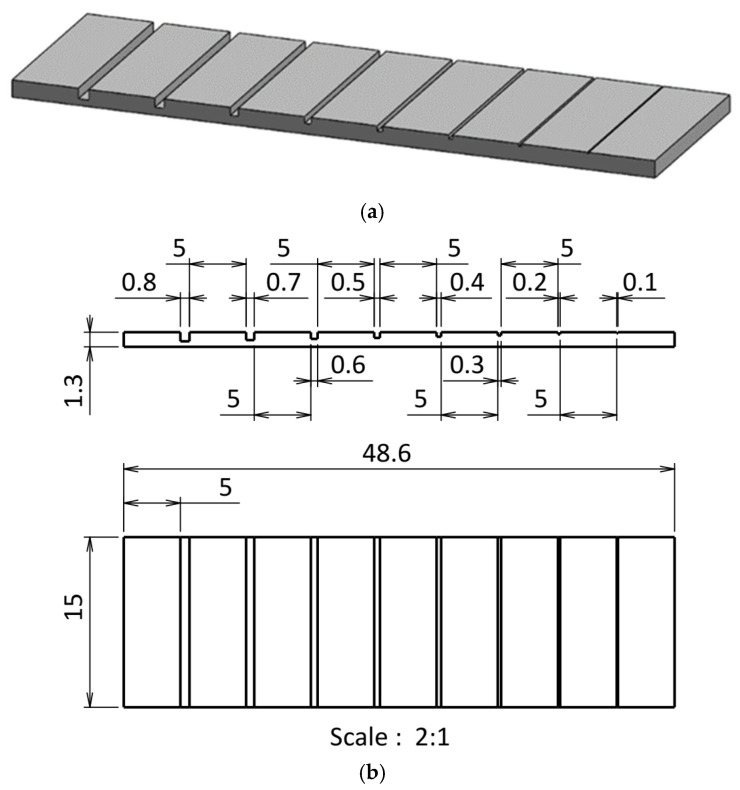
CAD design of the test part: (**a**) 3D model and (**b**) 2D.

The test part was designed using SolidWorks 2016 (Dassault Systèmes, Vélizy-Villacoublay, France) and exported in STL format for slicing. The part measures 48.6 mm × 15 mm × 3 mm (length × width × height), ensuring compatibility with common FDM build plates while minimizing print time and material usage. As illustrated in Figure 1, the design incorporates longitudinally aligned microchannels with nominal square cross-sections ranging from 0.8 mm to 0.1 mm in both width and depth, with decrements of 0.1 mm. Equal spacing between channels was implemented to prevent thermal coupling or mechanical distortion during printing.

An open-channel configuration was adopted to facilitate direct post-print optical inspection and to reflect typical surface-based microfluidic layouts. This structure enables efficient visual and quantitative evaluation of minimum printable feature sizes and serves as a repeatable basis for statistical comparisons across materials and parameter settings.

All test specimens were fabricated using a SHAREBOT 43 dual-extrusion FDM printer (Sharebot S.r.l., Nibionno, Italy), equipped with a 0.4 mm brass nozzle, a heated build plate (up to 120 °C), and independent dual extruders.

The 0.4 mm brass nozzle is the standard dimension for FDM printing and offers a practical compromise between extrusion stability and attainable feature resolution. This choice was made to maintain comparability with previous FDM microfluidics studies and to avoid introducing nozzle size as an additional variable in the Taguchi DOE. While smaller nozzles (e.g., 0.2 mm) could potentially improve lateral resolution, they significantly increase the risk of clogging and inconsistent flow with flexible TPU. The fixed 0.4 mm nozzle thus reflects a trade-off between resolution and process reliability.

The system supports layer heights as low as 80 µm, extrusion temperatures up to 320 °C, and a build volume of 300 × 250 × 200 mm. Its technical capabilities align with the demands of microscale feature resolution and experimental reproducibility.

Slicing was performed using SHAREBOT Print Slicer, which provides fine control over nozzle temperature, flow rate, infill strategies, and build orientation. After importing the STL files, G-code instructions were generated for each trial.

To ensure that the effects observed could be attributed to the parameters under investigation, a uniform set of fixed printing conditions was applied throughout all experiments. These include constant layer height, infill pattern, number of perimeters, and fan settings, as detailed in Table 1.

### 2.3. Experimental Procedure

To optimize FDM process parameters for microscale microchannel fabrication and assess their impact on dimensional accuracy, a structured Design of Experiments (DOE) approach was implemented using the Taguchi method [46]. The objective was to identify the most influential process parameters and determine their optimal settings for producing high-fidelity microchannels with TPU and PETG filaments.

A Taguchi L27 orthogonal array was selected to evaluate five process parameters at three levels each. This design enabled efficient analysis of main effects and variability using only 27 experimental conditions, as opposed to the 243 (3^5^) trials required by a full factorial design.

The five FDM parameters were selected based on their well-established influence on melt flow behavior, interlayer bonding, and geometrical precision at the microscale, as consistently reported in previous investigations. In addition, preliminary printability trials in our laboratory were used to confirm the practical feasibility of the selected ranges and to exclude extreme values that led to under-extrusion, unstable flow, or part warping. This dual basis, the literature precedent and empirical validation, ensured that the selected ranges were scientifically grounded and experimentally reliable.

For each material, the three levels of every parameter were defined within the material-specific thermal and processing envelope to ensure stable extrusion while covering the operational range relevant to microscale fabrication. These envelopes were not taken from datasheets but instead derived from ranges consistently reported in academic literature and confirmed by initial calibration prints performed in this study. The selected parameters and their levels are summarized in Table 2 (PETG) and Table 3 (TPU).

Nozzle temperature was set between 210 and 230 °C for TPU and 230 to 250 °C for PETG. These ranges align with those commonly reported for TPU [33,44,47,48] and PETG [28,44,49] in prior FDM microfabrication studies, ensuring comparability with the state of the art while remaining within the stable processing window identified during preliminary trials. The lower bounds prevented under-extrusion, cold flow, and poor wall definition, while the upper limits remained below the threshold for excessive stringing, thermal degradation, or melt instability. These settings allowed analysis of melt viscosity effects and layer fusion, which are essential for maintaining dimensional accuracy at the microscale.

Bed temperature, maintained between 40 and 60 °C for TPU and 70 to 90 °C for PETG, was selected to guarantee first-layer adhesion and minimize warping. TPU, owing to its low glass transition temperature, required lower bed temperatures to avoid softening and deformation of the part base. In contrast, PETG, which has a higher glass transition temperature, necessitated elevated bed temperatures to suppress edge lifting and promote consistent interlayer adhesion.

The range of printing speeds (20–40 mm/s for TPU and 30–50 mm/s for PETG) was defined to balance feature resolution with process stability. TPU’s high melt viscosity and elastic recovery necessitated lower speeds to minimize post-deposition swelling and distortion. PETG, more rigid and stable in flow, could be processed at higher speeds, allowing for increased throughput without sacrificing microchannel definition.

Flow rate was adjusted between 90 and 110% for TPU and 95 to 105% for PETG to address the materials’ distinct extrusion behaviors. TPU often exhibited under-extrusion due to elastic deformation in the filament path and thus required higher flow rates to achieve complete channel formation. PETG, on the other hand, was susceptible to over-extrusion and feature blurring at elevated flow rates, so its upper bound was set conservatively to preserve feature clarity.

Finally, infill overlap was varied between 15 and 25% for both materials. This parameter is particularly significant for microfluidics, where insufficient overlap can lead to unsealed perimeters and leakage, while excessive overlap risks overfilling and partial channel occlusion. The selected range, widely validated in the literature for microfluidic applications, offered a practical compromise to ensure wall integrity and channel openness.

In the Taguchi design, factor levels are represented in coded form (1, 2, and 3) within the L27 orthogonal array. To avoid ambiguity, these coded levels were explicitly mapped to the actual values specific to each material, as presented in Table 2 and Table 3. All subsequent analyses, tables, and figures use the coded representation for consistency with the orthogonal array, while interpretation of the results must be made with reference to the corresponding actual values provided in these tables. The complete L27 orthogonal array layout employed in this study is shown in Table 4.

**Figure 2:**
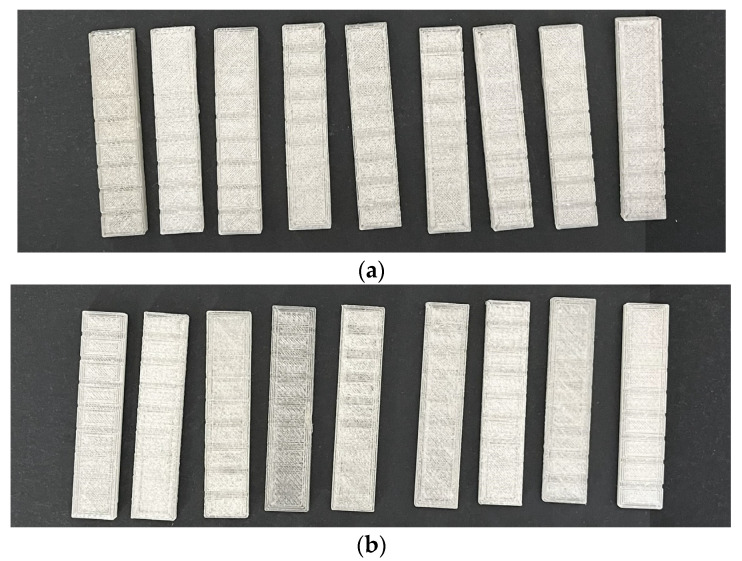
The printed samples from (**a**) PETG and (**b**) TPU.

Each of the 27 parameter combinations was printed in triplicate for each material, resulting in a total of 81 samples per material (162 total). 9 printed samples from PETG and TPU are presented in Figure 2.

The experimental sequence was randomized to mitigate systematic error, and all prints used the same standardized microchannel test part geometry and measurement protocols. This approach ensures that observed variations in output performance can be attributed directly to the controlled parameters rather than uncontrolled sources of variability.

### 2.4. Dimensional Measurement and Data Analysis

Following fabrication, each printed sample was inspected to evaluate the dimensional accuracy of the microchannel features. Channel width and depth were selected as the primary metrics, as both are critical for fluidic resistance and overall device functionality.

Measurements were performed using a Dino-Lite digital optical microscope (AnMo Electronics Corp., Taiwan) at 20× magnification, equipped with integrated measurement software. Prior to imaging, the microscope was calibrated with a traceable stage micrometer (10 µm divisions, ±1 µm accuracy) to ensure dimensional reliability.

Image analysis was carried out using the DinoCapture 2.0 software. To minimize user bias, channels were imaged orthogonally to the cross-section; oblique or distorted images were excluded from analysis.

For channel depth estimation, z-axis focusing was applied to locate the upper and lower channel boundaries, and depth was calculated from the calibrated focal plane displacement. This method provides an interpolated estimate of depth but does not imply control of sub-layer-height features, since the nominal Z-resolution of FDM is constrained by the selected layer height (0.1 mm in this study). Each measurement was repeated four times at different channel positions, and the reported values are presented as mean ± standard deviation to capture variability.

It should be noted that optical depth estimation in transparent PETG and compliant TPU is subject to systematic uncertainty due to light scattering, surface curvature, and elastic deformation during focusing. While the Dino-Lite system provides reproducible relative measurements, validation with contact-based profilometry or confocal microscopy would yield more accurate depth values.

Only channels that were fully formed and optically resolvable were included in the quantitative analysis. Features exhibiting severe over-extrusion, under-extrusion, incomplete walls, or clogging were classified as failed prints and excluded from dimensional quantification. The main failure modes were qualitatively observed and included perimeter delamination at high printing speeds, partial occlusion of channels under excessive overlap, and dimensional collapse of unsupported features in TPU. These observations provide useful context for interpreting the process limits.

To assess both accuracy and consistency, the Signal-to-Noise (*S*/*N*) ratio was employed as the principal performance metric. Based on the “smaller-is-better” criterion, the *S*/*N* ratio was calculated using the following equation:

(1) SN= −log10 1n∑i=1nyi2,

where $y_{i}$ represents the dimensional deviation for the *i*-th measurement, and n is the number of replicates. This formulation penalizes both large deviations and variability, making it suitable for evaluating microscale precision in printed features.

In addition to *S*/*N* analysis, an Analysis of Variance (ANOVA) was conducted to determine the statistical significance of each control factor and quantify its relative contribution to the total variance. The ANOVA was performed at a 95% confidence level using Minitab^®^ 21, providing *p*-values and percentage contributions for each factor. Interaction plots, main effect plots, and residual plots were also generated to explore parameter relationships and model robustness.

This analytical approach enabled the identification of optimal process parameters for both PETG and TPU, taking into account not only mean accuracy but also the consistency of feature reproduction across replicates. The combined use of *S*/*N* ratio analysis and ANOVA enabled the identification of material-specific optimal parameter sets, balancing dimensional accuracy with repeatability across all experimental conditions.

## 3. Results

### 3.1. Dimensional Accuracy

Dimensional accuracy of the printed microchannels was evaluated for both PETG and TPU by measuring width and depth deviations under all 27 process conditions defined by the Taguchi L27 orthogonal array.

For PETG, width deviations ranged from 0.0097 mm to 0.1709 mm, while depth deviations spanned 0.0093 mm to 0.1294 mm. These results reflect the complex interaction between thermal behavior, material flow properties, and the geometric constraints inherent to PETG at the microscale.

The highest dimensional fidelity was achieved in experiment N°17 at a nozzle temperature of 240 °C, bed temperature of 70 °C, printing speed of 30 mm/s, 100% flow rate, and 15% infill overlap, with both width and depth deviations minimized under these conditions. In contrast, the greatest dimensional losses were observed in experiment N°9 at a lower nozzle temperature (230 °C), higher bed temperature (90 °C), elevated flow rate (105%), and increased speed (40 mm/s), highlighting the detrimental impact of excessive material deposition and thermal accumulation on feature resolution. These findings underscore the importance of maintaining moderate extrusion temperatures and tightly controlled flow conditions for optimal PETG microchannel fabrication.

For TPU, dimensional deviations exhibited greater variability, reflecting the material’s intrinsic elasticity and its pronounced sensitivity to both flow rate and thermal environment. The optimal condition achieved in experiment 17 at 220 °C nozzle temperature, 60 °C bed temperature, 30 mm/s speed, 100% flow rate, and 15% overlap; yielded mean width and depth deviations of 0.0139 mm and 0.0006 mm, respectively. The poorest dimensional control occurred in experiment 9 at higher flow rates and printing speeds (notably at 110% flow and 40 mm/s), resulting in substantial over-extrusion and depth errors up to 0.0838 mm. Depth control in particular was compromised under high flow and low overlap settings, likely due to post-deposition swelling and insufficient stabilization time between layers. Moderate bed temperatures (50–60 °C) and printing speeds were found to favor dimensional stability, ensuring effective interlayer adhesion while avoiding thermal distortion.

Microscope cross-sectional images of PETG and TPU samples printed during experiments 9 and 17 are presented in Table 5.

A direct comparison reveals that PETG generally outperformed TPU in both dimensional fidelity and process robustness, attributable to its lower melt viscosity and greater structural rigidity. In contrast, TPU’s pronounced elastic behavior demands stricter regulation of flow and deposition parameters to minimize swelling and collapse in fine-featured geometries.

Collectively, these results confirm that nozzle temperature, flow rate, and printing speed are the dominant factors governing dimensional accuracy in FDM-printed microchannels, with material-specific sensitivities that must be addressed for reliable microfluidic device fabrication.

### 3.2. Statistical Analysis

#### 3.2.1. Signal-to-Noise Ratio Analysis

To evaluate the robustness of microchannel dimensional accuracy under varying process conditions, signal-to-noise (*S*/*N*) ratios were computed for each of the 27 experimental trials using the “smaller-the-better” criterion. This metric quantifies not only the average deviation from nominal dimensions but also the consistency of results across replicates and channel locations, providing a unified measure of process stability.

For PETG, *S*/*N* ratios for width deviation ranged from 10.43 to 39.43 dB, while depth *S*/*N* ratios spanned 14.92 to 39.70 dB, indicating considerable variability in robustness across the tested parameter space. Optimal robustness was consistently observed in parameter combinations featuring moderate nozzle temperatures, controlled flow rates, and moderate printing speeds, with experiment 17 yielding the highest *S*/*N* ratios for both width and depth. In contrast, lower *S*/*N* values were associated with high flow rates and increased printing speeds, reflecting greater susceptibility to process-induced errors and dimensional instability.

TPU exhibited a similar trend, though with overall lower *S*/*N* ratios and a narrower optimal window, reflecting its greater sensitivity to process fluctuations. Width *S*/*N* ratios ranged from 16.48 to 35.15 dB and depth *S*/*N* ratios ranged from 21.36 to 39.37 dB. As with PETG, the most robust conditions were achieved at moderate nozzle and bed temperatures, 100% flow rate, and moderate printing speeds, as evidenced by the results from experiment 17. Conversely, parameter combinations involving excessive flow or speed led to marked reductions in *S*/*N*, indicating a loss of dimensional control and increased variability, particularly pronounced in the flexible TPU matrix.

Across both materials, *S*/*N* ratio analysis consistently identified flow rate, nozzle temperature, and printing speed as the primary drivers of robustness in microchannel fabrication. The highest dimensional fidelity and repeatability were realized when these parameters were balanced, minimizing the influence of external disturbances and intrinsic process noise.

#### 3.2.2. Main Effects Analysis

**Figure 3:**
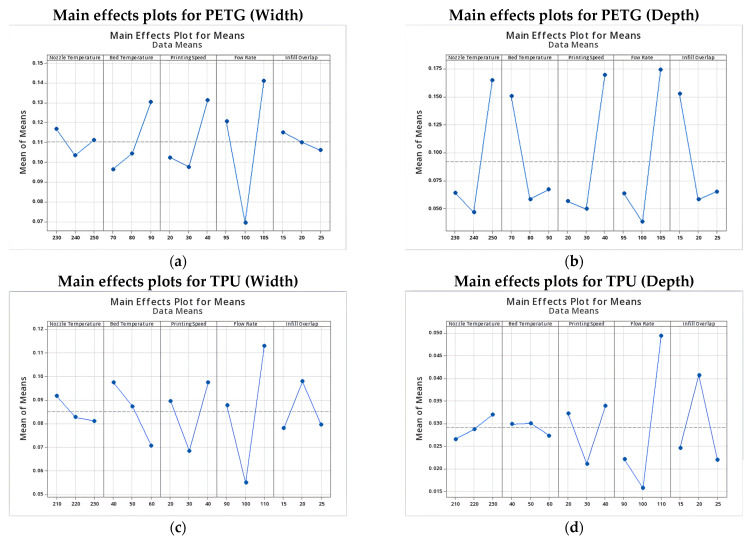
Main effects plots for PETG: (**a**) mean width deviation, (**b**) mean depth deviation, and TPU: (**c**) mean width deviation, and (**d**) mean depth deviation.

The analysis of the main effects plots, presented in Figure 3a–d, provides critical insight into how each process parameter individually influences the dimensional accuracy and robustness of microchannel fabrication in both PETG and TPU. The observed trends reveal distinct sensitivities for each material, reflecting differences in their rheological and thermal behavior.

For PETG, nozzle temperature, flow rate, and printing speed were identified as the most influential parameters. An optimal nozzle temperature of 240 °C yielded minimal dimensional deviations. Lower temperatures (230 °C) resulted in under-extrusion and poor layer fusion, while higher settings (250 °C) led to material softening and feature distortion. Flow rate exhibited the most pronounced effect: a setting of 100% ensured optimal control over both width and depth. Any deviation from this balanced value, whether due to over-extrusion (105%) or under-extrusion (90–95%), significantly impaired feature fidelity, particularly in narrow channels. Printing speed further modulated dimensional accuracy: a moderate speed of 30 mm/s offered the best trade-off between resolution and process stability. In contrast, higher speeds (40 mm/s) increased the risk of deposition instability and undersized features. Bed temperature and infill overlap played secondary roles; however, maintaining the bed temperature between 70 and 80 °C and applying a moderate infill overlap (15–20%) slightly improved wall definition and surface quality.

In TPU, similar trends were observed but with amplified sensitivities. The optimal nozzle temperature was identified as 220 °C; above this threshold, excessive softening and feature deformation became pronounced, particularly along the vertical axis. Flow rate again emerged as the dominant parameter, with 100% representing a critical equilibrium point. Higher rates (110%) induced lateral bulging, and interlayer swelling, whereas lower rates (90%) resulted in incomplete walls and underfilled channels. Printing speed had a marked effect: a speed of 30 mm/s consistently minimized dimensional deviations. Both slower (20 mm/s) and faster (40 mm/s) speeds increased dimensional errors, reflecting the delicate thermal and kinematic balance required for elastomeric materials. Bed temperature, optimally set at 60 °C, supported first-layer adhesion and build stability. Infill overlap exhibited a non-linear influence: the best performance was observed at 25%, followed closely by 15%, while intermediate levels yielded greater variability.

Overall, the main effects analysis highlights flow rate, nozzle temperature, and printing speed as the dominant factors governing dimensional fidelity and robustness in both PETG and TPU. However, the magnitude of parameter sensitivity is notably greater in TPU, which underscores the need for precise process control when fabricating microfluidic features from flexible polymers.

#### 3.2.3. Two-Factor Interactions Analysis

**Figure 4:**
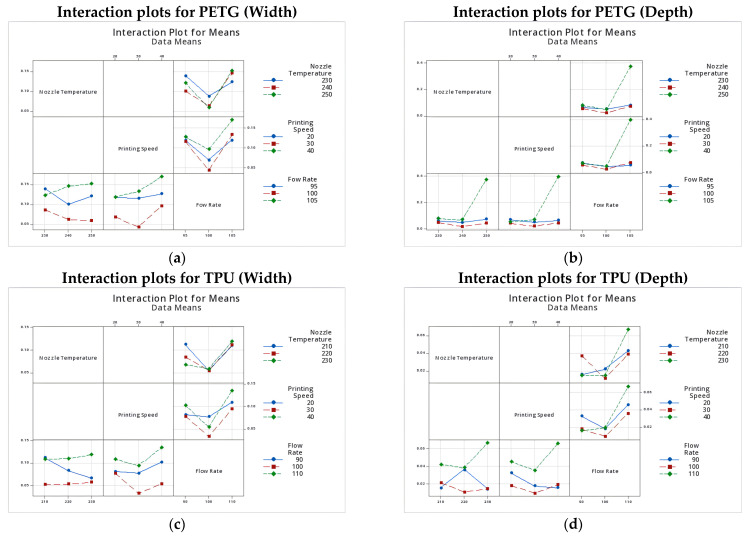
Interaction effects plots for PETG: (**a**) mean width deviation, (**b**) mean depth deviation, and TPU: (**c**) mean width deviation, and (**d**) mean depth deviation.

Beyond the influence of individual process parameters, the analysis of two-factor interactions reveals how the combined effects of key variables shape dimensional accuracy and robustness in microchannel fabrication. Interaction plots for mean deviation and signal-to-noise (*S*/*N*) ratio (Figure 4a–d) offer critical insight into the non-linear dependencies between parameters and the sensitivity of each material to process coupling.

For PETG, the interaction between nozzle temperature and flow rate was particularly pronounced. At the optimal nozzle temperature of 240 °C, dimensional deviations remained low and stable across all tested flow rates, indicating a robust processing window.

However, at lower temperatures (230 °C), increasing the flow rate to 105% resulted in a substantial loss of dimensional fidelity, likely due to insufficient melt viscosity combined with over-extrusion. At the opposite extreme, the highest nozzle temperature (250 °C) combined with elevated flow rates exacerbated depth errors, suggesting material softening and overflow into the channel voids. The interaction between printing speed and flow rate also showed a significant effect: pairing a 100% flow rate with a moderate speed of 30 mm/s consistently minimized deviations, whereas the combination of high speed (40 mm/s) and high flow (105%) produced marked dimensional instability, particularly in the depth axis.

In TPU, interaction effects were similarly significant but often more pronounced, reflecting the material’s greater sensitivity to process variations. The nozzle temperature × flow rate interaction emerged as the dominant factor. Dimensional accuracy and robustness peaked at 220 °C combined with 100% flow rate, with both mean deviation and *S*/*N* ratio showing minimal sensitivity to process fluctuations under these conditions. In contrast, the combination of elevated temperature (230 °C) and excessive flow rate (110%) resulted in a rapid degradation of both width and depth fidelity, along with increased variability. This behavior is characteristic of thermal over-softening and material flooding, commonly observed in elastomeric prints. The interaction between printing speed and flow rate further highlighted the delicate balance required for TPU processing: optimal results were achieved at 30 mm/s and 100% flow, while higher speeds, when combined with increased flow, quickly undermined dimensional control, particularly in the vertical (depth) direction.

The combined analysis of two-factor interactions emphasizes the critical importance of synchronizing extrusion and motion parameters, particularly flow rate and temperature, to achieve robust and repeatable microchannel features. PETG exhibits a broader tolerance to parameter coupling, whereas TPU requires tighter control to prevent the amplification of errors and process instability. These insights are essential for defining robust process windows and ensuring the practical viability of FDM-based microfluidics for both rigid and flexible device platforms.

#### 3.2.4. ANOVA Analysis

To statistically assess the influence of process parameters on microchannel dimensional accuracy and repeatability, a one-way analysis of variance (ANOVA) was conducted for both PETG and TPU, considering mean deviations and standard deviations in both width and depth. The ANOVA results quantify the relative contributions of each parameter and support the significance of the trends identified in the main effects and interaction analyses.

For PETG, the ANOVA on mean width deviation (Table 6) revealed a highly significant effect of the process parameters (F = 42.304, *p* < 0.001), confirming the findings from the main and interaction plots. In particular, flow rate, printing speed, and nozzle temperature were identified as the most influential parameters affecting horizontal dimensional fidelity. The strength of this effect underscores the importance of parameter tuning in minimizing width deviation and achieving dimensional accuracy.

Similarly, the ANOVA on mean depth deviation (Table 7) showed a statistically significant response (F = 12.134, *p* ≈ 1.3 × 10^−5^), indicating that depth accuracy, although often constrained by machine-specific characteristics such as z-axis resolution, can still be effectively influenced by extrusion and thermal conditions. Specifically, bed temperature, nozzle temperature, and flow rate were found to govern depth accuracy by affecting material sagging, over-deposition, and underfill within the channels. This result highlights that vertical precision is not solely hardware-limited but is also responsive to process parameter control.

For TPU, the ANOVA confirmed the statistically significant effect of process parameters on mean width deviation (F = 7.378, *p* = 0.00039), as shown in Table 8. This suggests that dimensional accuracy in elastomeric materials is particularly sensitive to tuning of nozzle temperature, bed temperature, printing speed, flow rate, and infill overlap. Likewise, the model for mean depth deviation (Table 9) also exhibited a significant response (F = 7.883, *p* = 0.00026), indicating that vertical dimensional precision is uneven across the design space and is notably affected by processing conditions.

These results provide dual confirmation that, for TPU, both width and depth deviations are significantly influenced by parameter variation. They underscore the critical importance of precise parameter optimization when working with flexible filaments, where thermal and mechanical behavior is more complex and less forgiving than with rigid materials.

**Figure 5:**
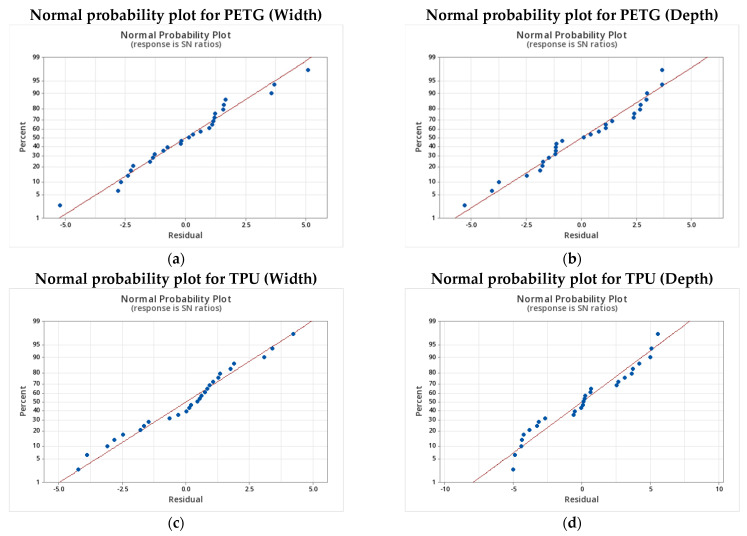
Normal probability plot for PETG: (**a**) width, (**b**) depth, and for TPU: (**c**) width, and (**d**) depth.

To verify the validity of the Taguchi model and the assumptions underlying the ANOVA results, a residual analysis was conducted. Normal probability plots of the residuals for both width and depth signal-to-noise (*S*/*N*) ratios are shown in Figure 5a–d.

In both PETG and TPU, the residuals closely followed a normal distribution, indicating the absence of model misspecification or substantial deviation from normality. This supports the assumption of homoscedasticity and justifies the use of parametric statistical techniques, thereby reinforcing the robustness and reliability of the conclusions drawn from the ANOVA.

Taken together, these results demonstrate that the careful selection and tuning of FDM process parameters can have a statistically validated and substantial impact on the dimensional accuracy and repeatability of microchannel fabrication. This is particularly evident in the control of width deviation across both materials, as well as depth fidelity in flexible polymers such as TPU. The findings provide a strong foundation for targeted process optimization and underscore the importance of material-specific parameter mapping in advancing the precision and applicability of additive manufacturing for microfluidic devices.

### 3.3. Synthesis

The experimental findings clearly establish that the optimization of FDM process parameters is essential for achieving precise and reliable microchannel fabrication. Significant differences in sensitivity and performance were observed between PETG and TPU, reflecting their distinct material characteristics and process responses.

For PETG, the combined analysis of dimensional deviations, standard deviations, and signal-to-noise ratios identifies nozzle temperature, flow rate, and printing speed as the dominant parameters governing microchannel accuracy. Optimal performance was attained at a nozzle temperature of 240 °C, a 100% flow rate, and a printing speed of 30 mm/s. These conditions provided a favorable balance between material flow, thermal stability, and deposition precision. Under these optimized settings, PETG consistently enabled the fabrication of microchannels with widths as small as 100 µm, establishing a practical lower limit for lateral resolution in PETG-based microfluidic devices. Depth values close to the nominal 100 µm design were estimated optically, though constrained by the 0.1 mm layer height, and should therefore be interpreted as approximations rather than true sub-layer-height capability. Although both mean width and depth deviations improved significantly through parameter tuning, depth repeatability exhibited lower responsiveness. This limitation is attributable not only to hardware constraints such as the fixed 0.1 mm layer height and z-axis precision, but also to the inherent uncertainties of optical depth estimation in transparent and flexible polymers. Depth values were derived from z-axis focusing, which provides reproducible but approximate interpolations of surface boundaries. Future studies employing profilometry or confocal microscopy would enable more rigorous validation.

In contrast, TPU exhibited greater sensitivity to process fluctuations, particularly due to its elasticity and post-deposition swelling behavior. Among the studied parameters, flow rate and printing speed had the most significant impact on dimensional accuracy. Optimal control was achieved at intermediate printing speeds (30 mm/s) and moderate flow rates (100%), under which microchannel widths below 150 µm were achievable, provided that conditions were precisely regulated. Statistical analysis confirmed the influence of process parameters not only on mean dimensional deviations but also on depth variability, while lateral repeatability remained relatively stable. Despite these improvements, matching the dimensional robustness of PETG remains challenging with TPU, emphasizing the necessity for tighter process control and more detailed parameter mapping in flexible material printing. In TPU, depth measurements must also be interpreted with caution, as light scattering, surface curvature, and elastic deformation during focusing introduce additional uncertainty.

Taken together, these findings offer actionable guidance for the selection and tuning of FDM process parameters based on the target material system and feature resolution. PETG provides a broad and reliable processing window for microscale applications, whereas TPU, although capable of fine resolution, demands more stringent optimization to achieve comparable precision and repeatability. These insights establish a solid foundation for the advancement of FDM-based microfluidic device fabrication across both rigid and flexible platforms, while also highlighting the need for further studies incorporating nozzle diameter variation and contact-based depth validation to fully define the resolution limits of the technique.

## 4. Conclusions

This study systematically investigated the dimensional accuracy and process robustness of PETG and TPU for the fabrication of microscale channels using FDM. Using a Taguchi design of experiments and statistical analysis, nozzle temperature, flow rate, and printing speed were identified as the dominant factors governing feature fidelity.

PETG enabled reproducible fabrication of microchannels as small as 100 µm, establishing a practical resolution threshold for rigid and transparent microfluidic devices such as optical detection chips, chemical sensors, and lab-on-chip diagnostic platforms. TPU, while more variable, supported sub-150 µm features under optimized conditions, making it suitable for flexible and wearable microfluidics, including soft pumps, stretchable sensors, and skin-mounted drug delivery patches. These distinct material-specific behaviors highlight the trade-offs between dimensional fidelity and functional adaptability, while also demonstrating the feasibility of integrating rigid and flexible elements within a single fabrication framework.

The contributions of this work can be summarized as follows:

- It introduces a systematic multi-factor optimization framework for FDM microfabrication, incorporating rarely studied parameters such as flow rate and infill overlap that are critical for channel sealing and leakage prevention, in addition to the commonly investigated printing speed and temperature.
- It demonstrates a dual-material optimization of PETG and TPU under identical microscale fabrication constraints, defining reproducible processing windows for both rigid and flexible microfluidic platforms and supporting their integration into hybrid rigid-flexible devices.
- It establishes the reproducible fabrication of sub-150 µm channels, with reliable features as small as 100 µm, thereby benchmarking the achievable resolution of desktop FDM against conventional fabrication approaches. While soft lithography and resin-based printing can achieve finer resolution, FDM uniquely combines affordability, accessibility, and material versatility for rapid and customizable prototyping.

By directly connecting optimized printing parameters with device-level feasibility, this study provides practical guidelines for researchers aiming to translate low-cost FDM into functional microfluidic systems. The results advance the development of accessible, application-driven devices ranging from diagnostic assays to wearable health monitors and reinforce the role of multi-material FDM as a viable alternative to conventional microfabrication for next-generation microfluidic platforms.

Preliminary multi-material printing trials provided qualitative indications of promising adhesion between PETG and TPU, enabling the fabrication of integrated rigid–flexible structures. Since these bonding observations were not quantified with dedicated adhesion tests, they are presented here as exploratory insights rather than validated outcomes. Nevertheless, the absence of spontaneous delamination during handling and post-processing suggests that reliable inter-material interfaces may be achievable with optimized conditions. Future work will therefore include systematic adhesion characterization (e.g., peel or tensile tests) to confirm interface strength and durability, as well as the fabrication of fully functional multi-material microfluidic devices that combine PETG’s transparency and structural rigidity with TPU’s elasticity and compliance. Such devices will be evaluated in practical contexts, for example, in wearable drug delivery or health-monitoring platforms, where hybrid rigid-flexible architectures are essential for combining stable reservoirs with conformable skin-contact interfaces. This progression from parameter optimization to application-oriented validation will further demonstrate the potential of low-cost FDM as a versatile platform for decentralized and customizable microfluidic manufacturing.

## Figures and Tables

**Table 1:** Fixed printing parameters used in all experiments.

Parameter	Value	Justification
Layer height	0.1 mm	Balances vertical resolution and build time
Infill density	20%	Balances vertical resolution and build time
Infill orientation	45°	Provides uniform internal support and consistent surface quality
Build orientation	Flat on the XY plane	Open channels facing upward to facilitate geometry resolution and measurements

**Table 2:** Factors with corresponding levels for PETG.

Parameters	Level 1	Level 2	Level 3
Nozzle Temp (°C)	230	240	250
Bed Temp (°C)	70	80	90
Printing Speed (mm/s)	30	40	50
Infill overlap (%)	15	20	25
Flow Rate (%)	95	100	105

**Table 3:** Factors with corresponding levels for TPU.

Parameters	Level 1	Level 2	Level 3
Nozzle Temp (°C)	210	220	230
Bed Temp (°C)	40	50	60
Printing Speed (mm/s)	20	30	40
Infill overlap (%)	15	20	25
Flow Rate (%)	90	100	110

**Table 4:** The standard layout of the L27 orthogonal array.

Experiment	Nozzle Temperature	Bed Temperature	Printing Speed	Flow Rate	Infill Overlap
1	1	1	1	1	1
2	1	1	1	2	2
3	1	1	1	3	3
4	1	2	2	1	1
5	1	2	2	2	2
6	1	2	2	3	3
7	1	3	3	1	1
8	1	3	3	2	2
9	1	3	3	3	3
10	2	1	2	1	2
11	2	1	2	2	3
12	2	1	2	3	1
13	2	2	3	1	2
14	2	2	3	2	3
15	2	2	3	3	1
16	2	3	1	1	2
17	2	3	1	2	3
18	2	3	1	3	1
19	3	1	3	1	3
20	3	1	3	2	1
21	3	1	3	3	2
22	3	2	1	1	3
23	3	2	1	2	1
24	3	2	1	3	2
25	3	3	2	1	3
26	3	3	2	2	1
27	3	3	2	3	2

**Table 5:** Microscope cross-sectional images of PETG and TPU samples.

Material	Exp N°	Microscope Images of Printed Samples		
PETG	17	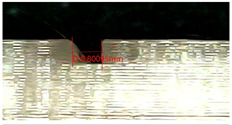	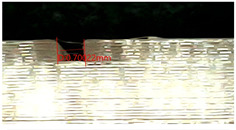	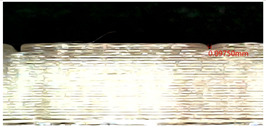
	9	** 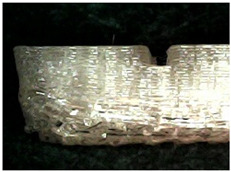 **	** 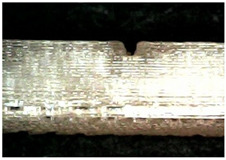 **	** 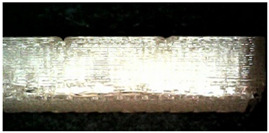 **
TPU	17	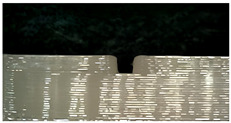	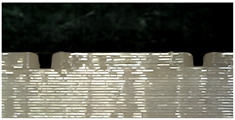	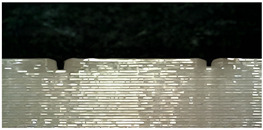
	9	** 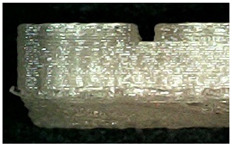 **	** 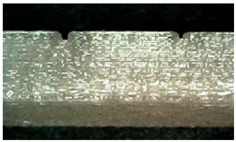 **	** 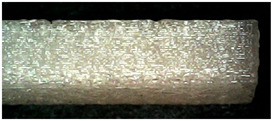 **
Scale Bar		** 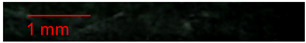 **		

**Table 6:** ANOVA for mean width deviation for PETG.

	SumSq	DF	MeanSq	F	*p*-Value
Total	0.552	26	0.0212		
Model	0.503	5	0.1006	42.304	2.888 × 10^−10^
Residual	0.049	21	0.0023		

**Table 7:** ANOVA for mean depth deviation for PETG.

	SumSq	DF	MeanSq	F	*p*-Value
Total	0.269	26	0.103		
Model	0.2003	5	0.04	12.134	1.2993 × 10^−5^
Residual	0.069	21	0.0033		

**Table 8:** ANOVA for mean width deviation for TPU.

	SumSq	DF	MeanSq	F	*p*-Value
Total	0.198	26	0.0076		
Model	0.126	5	0.0252	7.3778	0.00039
Residual	0.071	21	0.0034		

**Table 9:** ANOVA for mean depth deviation for TPU.

	SumSq	DF	MeanSq	F	*p*-Value
Total	0.0325	26	0.0012		
Model	0.0212	5	0.0042	7.8835	0.00025
Residual	0.0113	21	0.00053		

## Data Availability

The original contributions presented in this study are included in the article. Further inquiries can be directed to the corresponding author.
